# Clinical Validation of Type 1 Diabetes Coding in Hospital Discharge Records Using ADA Criteria: Implications for Spanish and European Health Data Spaces

**DOI:** 10.3390/jcm15062286

**Published:** 2026-03-17

**Authors:** Rafael Gómez-Coronado-Martín, Miguel Ángel Salinero-Fort, Ana López-de-Andrés, Daniala L. Weir, Carmen de Burgos-Lunar

**Affiliations:** 1 Servicio de Admisión y Documentación Clínica, Hospital Clínico San Carlos, Instituto de Investigación Sanitaria del Hospital Clínico San Carlos (IdISSC), 28040 Madrid, Spain; rafael.gomezcoronado@salud.madrid.org; 2 Department of Public Health and Maternal & Child Health, Universidad Complutense de Madrid, Instituto de Investigación Sanitaria del Hospital Clínico San Carlos (IdISSC), 28040 Madrid, Spain; anailo04@ucm.es (A.L.-d.-A.); carmen.deburgos@salud.madrid.org (C.d.B.-L.); 3 Division of Pharmacoepidemiology & Clinical Pharmacology, Utrecht Institute for Pharmaceutical Sciences (UIPS), Utrecht University, 3584 CS Utrecht, The Netherlands; d.l.weir@uu.nl; 4 Network for Research on Chronicity, Primary Care, and Health Promotion (RICAPPS), Carlos III Health Institute, 28029 Madrid, Spain; 5 Frailty, Multimorbidity Patterns and Mortality in the Elderly Population Residing in the Community—Hospital La Paz Institute for Health Research IdiPAZ, 28046 Madrid, Spain; 6 Servicio de Medicina Preventiva, Hospital Clínico San Carlos, Instituto de Investigación Sanitaria del Hospital Clínico San Carlos (IdISSC), 28040 Madrid, Spain

**Keywords:** type 1 diabetes mellitus, CMBD, validation study, hospital discharge data, epidemiology

## Abstract

**Background/Objectives**: Administrative and clinical databases are increasingly used for research, but their value depends on coding accuracy. The Spanish National Hospital Discharge Database (CMBD) is a standardised registry widely applied in epidemiology. Type 1 diabetes mellitus (T1DM) is an autoimmune disease with early onset and long-term complications. This study aimed to validate the accuracy of T1DM diagnoses recorded in the CMBD. **Methods**: A cross-sectional validation study was conducted at Hospital Clínico San Carlos (Madrid, Spain) including discharges from 2016–2023. Two age- and sex-matched samples of 384 admissions each (with and without T1DM coding, ICD-10 E10) were randomly selected. The gold standard was the confirmation of T1DM based on the diagnostic criteria established by the 2016 American Diabetes Association (ADA) consensus, which remained valid through 2025, verified by a detailed review of electronic health records (EHRs). Sensitivity, specificity, positive predictive value (PPV), and negative predictive value (NPV) were calculated with 95% confidence intervals (CIs), and interobserver concordance was assessed with Cohen’s kappa. **Results**: Of the 245,206 discharges, 1324 (0.54%) included a T1DM diagnosis. Validation showed a sensitivity of 100% (95% CI: 98.7–100), specificity of 80.2% (95% CI: 76.4–83.5), PPV of 75.3% (95% CI: 70.7–79.3), and NPV of 100% (95% CI: 99.0–100). Interobserver agreement was excellent (κ = 0.869). Specificity declined with age, from 100% in patients < 30 years to 60% in those ≥ 80 years, mainly due to misclassification with insulin-treated type 2 diabetes. **Conclusions**: T1DM diagnoses in the CMBD show very high validity and reliability in younger patients, supporting their use in epidemiological and clinical research, while complementary verification is advisable in older adults.

## 1. Introduction

Type 1 diabetes mellitus is a chronic autoimmune disease in which T lymphocytes destroy pancreatic β-cells that produce insulin, leading to an absolute and permanent insulin deficiency [1]. Although no significant impact on quality of life is usually observed in childhood [2], this impact increases during adolescence and youth [3,4], and becomes even more pronounced in adulthood, when severe chronic complications begin to appear. Among these, cardiovascular diseases represent the main cause of morbidity and mortality [5]. In addition, T1DM is associated with reduced life expectancy [5,6] and with substantial costs for both individuals and healthcare systems, particularly in terms of direct costs [7,8].

Spain is among the countries with the highest reported prevalence of T1DM. In 2021, an estimated 203,865 people were living with T1DM, corresponding to a prevalence of 428 per 100,000 inhabitants (186 per 100,000 in those < 20 years and 486 per 100,000 in adults ≥ 20 years) [6]. When diagnosis occurs at age 10, life expectancy is reduced by approximately 6 years [6]. Given the early onset, clinical complexity, and long-term comorbidity burden of T1DM, reliable registries that accurately identify affected individuals are essential.

Administrative and clinical databases are increasingly used to generate evidence on effectiveness and safety under real-world conditions and at scale [9,10,11]. However, their validity depends on the accuracy and completeness of recorded data. Misclassification may arise from the incomplete capture of key variables or from errors in documentation and coding. Notably, code validity is seldom assessed: only 12% of studies reported the verification of diagnostic accuracy, and in some contexts, the probability that a positive code reflects a true diagnosis may be below 50% [12]. Data quality therefore depends not only on the coding system (e.g., ICD-10 or SNOMED CT), but also on the broader information pathway such as healthcare access, clinical assessment and documentation practices, and institutional coding and reimbursement procedures that ultimately determines what is recorded and coded [13].

The Minimum Basic Dataset of Hospital Discharges (CMBD) is a nationwide clinical–administrative database that has been mandatory since 1992 for all hospitals in the Spanish National Health System and was extended to private hospitals in 2005. It captures all hospital discharges using a standardised structure and uniform coding, including demographic characteristics, admission and discharge information, primary and secondary diagnoses coded according to ICD-10, and diagnostic and therapeutic procedures. Diagnoses and procedures are coded by trained professionals in admission and clinical documentation services, applying unified national criteria issued by the Ministry of Health and the Technical Unit for Clinical Classifications. As with any classification process, coding may generate false positives and false negatives, which can affect case ascertainment and bias estimates derived from administrative data [14].

Beyond its clinical relevance, the accurate identification of individuals with type 1 diabetes in electronic health records has become increasingly important in the context of emerging national (HealthData MAD-R&I) and European health data (HealthData@EU) infrastructures. The Spanish National Health Data Space [15] and the proposed European Health Data Space (EHDS) explicitly require high-quality, interoperable, and clinically validated data to enable secure secondary use for research, public health, and policy evaluation [16]. Reliable diagnostic coding is therefore essential to ensure that hospital discharge databases such as the CMBD can be safely integrated into these federated data ecosystems and used for large-scale analytics [17].

Despite the clinical importance of T1DM and its burden in Spain, hospital CMBD diagnoses of T1DM have not been directly validated using clinically verified reference criteria. This study therefore aims to validate T1DM diagnoses recorded in the hospital CMBD to support the reliability of future epidemiological and clinical research using these data.

## 2. Materials and Methods

### 2.1. Study Design

A retrospective cross-sectional validation study was conducted on T1DM diagnoses recorded in the CMBD, using the 2016 ADA diagnostic criteria as the gold standard [18].

### 2.2. Setting

The study was conducted at Hospital Clínico San Carlos (HCSC), a tertiary-care hospital in the Community of Madrid, which serves a population of more than 377,000 individuals. HCSC comprises 861 beds and 24 operating rooms and provides care to a reference population of 377,831 inhabitants, representing 10.58% of the population of the city of Madrid and 5.17% of that of the Autonomous Community of Madrid [19].

### 2.3. Population

The study was based on selecting the population discharged from inpatient care at Hospital Clínico San Carlos during the period 2016–2023. From this population, two samples were identified: the first included patients with at least one record of T1DM (ICD-10 codes E10–E14) in the electronic health records, and the second comprised patients without this diagnosis in the electronic health records. The first sample was defined as probable cases, and the second as probable non-cases. In both samples, admissions were excluded if no plasma glucose measurement, either fasting or random, was documented in the EHR.

### 2.4. Data Sources

T1DM diagnoses recorded in the CMBD were validated against the American Diabetes Association (ADA) criteria, which are described in detail in Section 2. To assess these criteria, multiple electronic data sources were consulted, including hospital health records, primary care records, and outpatient clinic records from Hospital Clínico San Carlos. A direct validation approach was employed by reviewing the individual clinical records of each patient across care settings to confirm adherence to diagnostic criteria.

### 2.5. Sample

The selection of diagnoses was carried out using the ICD-10 code for T1DM (ICD-10 E-10) during the established period. E10 was selected because it is the specific code for type 1 diabetes in the Spanish CMBD and is used for clinical, administrative, and reimbursement purposes when applicable. Given the absence of reference data on the expected proportion of misclassifications (false negatives or false positives), we assumed the maximum indeterminacy scenario (*p* = q = 0.5). Based on this assumption, and for 95% confidence and 5% precision, a sample of 384 patients was required.

Two patient samples were obtained to validate T1DM episodes: 384 MHs with ICD-10 E-10 codes (sample 1) and 384 MHs without this code (sample 2), resulting in a total sample of 768 admissions. Since the probability of presenting these episodes is related to age and their distribution differs between sexes, the sample of patients without disease codes were matched to the coded sample by age and sex.

Admissions with T1DM coding were first identified from the CMBD database using ICD-10 code E10 and its corresponding specific subcodes recorded in the principal and secondary diagnosis fields. From this source population, participants in sample 1 were selected by simple random sampling. Sample 2 was drawn from admissions without T1DM coding in the same CMBD source population and matched 1:1 to the coded sample by sex and age.

### 2.6. Methods

Diagnostic tests aim to correctly classify individuals according to whether they present with a disease or clinical condition. Their validation is based on comparing the results obtained through the test with those of a reference instrument considered the most reliable standard, the so-called gold standard.

For validation purposes, T1DM coding in hospital discharge reports was conceptualised as a diagnostic test. We evaluated its diagnostic performance by comparing it with a clinical gold standard based on ADA criteria.

The diagnostic criteria used for validation were based on the 2025 American Diabetes Association (ADA) consensus statement. Although this consensus statement was published in 2025, no changes had been made to the diagnostic standards for T1DM since the start of the study period in 2016. These diagnostic criteria also reflect the standards used in Spain for the clinical diagnosis and classification of T1DM. They include the standard diagnostic thresholds for diabetes: A1C ≥ 6.5% (48 mmol/mol), fasting plasma glucose ≥ 126 mg/dL (7.0 mmol/L), 2-h plasma glucose ≥ 200 mg/dL (11.1 mmol/L) during a 75 g oral glucose tolerance test, or a random plasma glucose ≥ 200 mg/dL (11.1 mmol/L) in the presence of classic symptoms. Distinction from type 2 diabetes is based on the presence of autoimmune markers (islet autoantibodies), age at onset, clinical phenotype, and the need for insulin therapy. The ADA 2025 further classifies T1DM into three stages: stage 1 (multiple autoantibodies, normoglycaemia), stage 2 (autoantibodies with dysglycaemia), and stage 3 (symptomatic diabetes with overt hyperglycaemia) [18].

**Figure 1:**
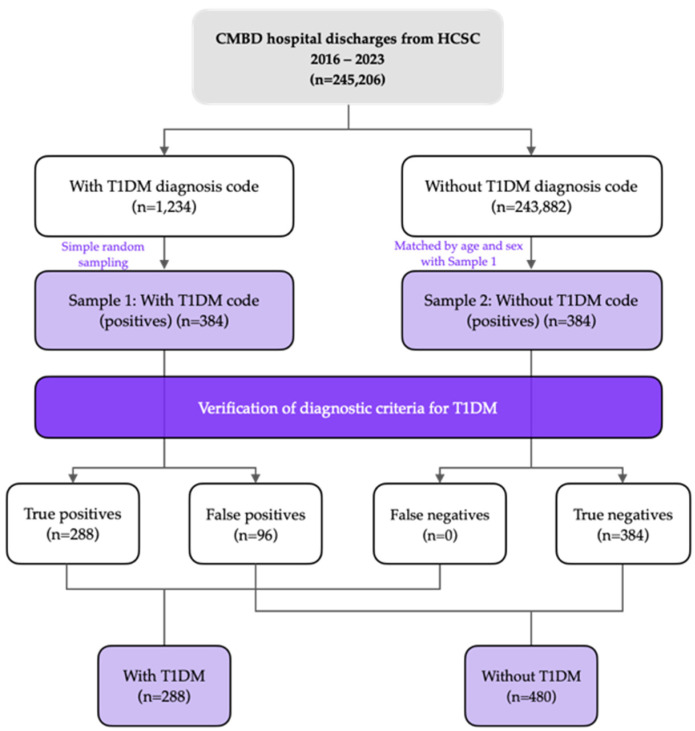
Flowchart of the study design and validation process of type 1 diabetes mellitus (T1DM) diagnoses in the Spanish National Hospital Discharge Database (CMBD) at Hospital Clínico San Carlos, 2016–2023.

The validation algorithm used is shown in Figure 1.

Validation was conducted through structured manual review of the EHR for each sampled admission, applying predefined ADA-based clinical and diagnostic criteria uniformly to all cases. Two independent physician-epidemiologists, blinded to the CMBD coding status, assessed whether diagnostic criteria for diabetes were fulfilled and whether the overall clinical profile supported classification as T1DM rather than another diabetes type. Reviews were performed in parallel, with disagreements resolved by consensus. Inter-rater agreement was quantified using Cohen’s kappa. The validation algorithm is presented in Figure 1. Patients and the public were not involved in the design, conduct, reporting, or dissemination plans of this research.

### 2.7. Statistical Analysis

A descriptive analysis of the study populations and samples was performed. Continuous variables were summarised using the mean and standard deviation, and the median and interquartile range were reported for skewed distributions. Categorical variables were expressed as relative frequencies.

Overall agreement between recorded diagnoses and the gold standard, as well as interobserver concordance, was assessed using Cohen’s kappa coefficient with 95% confidence intervals (CIs).

Sensitivity (Se), defined as the proportion of true positives correctly classified as T1DM cases, and specificity (Sp), defined as the proportion of true negatives correctly classified as non-cases, were calculated together with the positive predictive value (PPV) and negative predictive value (NPV), each with their 95% CIs. All measures were estimated, both overall and stratified by sex and age group.

A sensitivity analysis was conducted to determine the impact of varying disease prevalence on predictive values for T1DM. Homogeneity of sensitivity and specificity across sex and age strata was assessed using the χ^2^ test. When assumptions for this test were not met (i.e., any expected frequency < 5), Fisher’s exact bilateral test was applied.

All estimates were reported with 95% CIs. The significance level for hypothesis testing was set at 0.05. Statistical analyses were performed using STATA BE 17^®^ and IBM SPSS Statistics 26^®^. Confidence intervals for kappa and predictive values were obtained with the SPSS macros !KAPPA and !DT, developed by the Applied Statistics Laboratory at the Autonomous University of Barcelona [20].

## 3. Results

### 3.1. Population Characteristics

Between 2016 and 2023, a total of 245,206 hospital discharges were recorded at HCSC, of which 1324 (0.54%) included a diagnosis coded as T1DM.

The main characteristics of patients in hospitalisation episodes, overall, in those with T1DM, and in the selected sample, are summarised in Table 1. No statistically significant differences were observed between all patients with a T1DM diagnosis and those included in the validation sample.

Patients admitted with T1DM were younger than the general hospital population, with a mean age of 51.2 years compared with 62.4 years, and the proportion of women was lower among patients with T1DM (45.8%) than among all hospitalised patients (52.8%).

Compared with the total hospitalised population, those with T1DM showed a higher prevalence of chronic kidney disease and greater comorbidity burden (Charlson index), but lower prevalence of dementia and malignant neoplasms. No differences were found in mortality, acute myocardial infarction, or cerebrovascular disease.

Length of stay and 30-day readmission rates did not differ significantly between the two groups.

### 3.2. Specificity, Sensitivity, and Overall Agreement

Inter-observer concordance was high, with a kappa index of 0.869 (95% CI 0.831–0.906), indicating almost perfect agreement between evaluators [21].

Coding of T1DM in hospital discharge reports at HCSC showed a sensitivity of 100%, as no false negatives were identified in the analysed sample. Specificity was 80.3% (95% CI 74.28–85.18), with 24.7% false positives (95 cases). All false positives were due to hyperglycaemia from causes other than T1DM: in 93.7%, the patients had type 2 diabetes mellitus, most of them insulin-dependent; 2 patients (2.1%) had diabetes secondary to pancreatectomy; 2 patients had hyperglycaemia without diabetes; 1 had maturity-onset diabetes of the young (MODY); and 1 had cystic fibrosis.

The kappa index for diagnostic agreement between CMBD coding and ADA criteria was 0.753 (95% CI 0.707–0.798). Interobserver agreement for the adjudication of T1DM status was Cohen’s κ = 0.869 (95% CI, 0.831–0.906), indicating almost perfect agreement.

No differences in sensitivity were observed when stratified by sex and age group, as shown in Table 2. Specificity declined progressively with increasing age, from 100% in patients younger than 30 years to 60% (95% CI 49.04–70.04) in those aged 80 years or older. This trend was also reflected in the kappa indices for diagnostic agreement, which decreased progressively with age.

### 3.3. Sensitivity Analysis

As PPV is directly proportional to disease prevalence and NPV inversely proportional, these indicators were estimated for different actual prevalence values of both conditions using the sensitivity and specificity results obtained in our study (Table 3).

The NPV was 100% across all scenarios analysed due to the high sensitivity of the study and the low prevalence of T1DM. However, the probability of confirming diagnostic criteria in patients with a T1DM episode recorded in their EMR (PPV for a prevalence of 0.54) was 2.67% (95% CI 2.07–2.94).

In patients younger than 30 years, where no false positives or false negatives were detected, both PPV and NPV were 100% (Table 4).

## 4. Discussion

The study found that T1DM coding in the CMBD at Hospital Clínico San Carlos had a sensitivity of 100% and an NPV of 100%, indicating that not clinically confirmed T1DM cases were missed in the study sample. Specificity was 80.2%, and 24.7% of episodes coded as T1DM were not confirmed by the ADA-based clinical review, resulting in a PPV of 75.3%. Agreement between CMBD coding and the ADA-based reference assessment yielded κ = 0.753, and interobserver concordance for the reference assessment was κ = 0.869, supporting consistency between evaluators.

Specificity varied across age groups, declining from 100% in patients younger than 30 years to 60% in those aged 80 years or older, with a parallel decrease in kappa. This pattern is compatible with differences in diagnostic distinctiveness across the life course. T1DM is most often diagnosed at younger ages and may follow more clearly documented diagnostic trajectories, whereas at older ages, misclassification with insulin-treated T2DM becomes more likely, particularly when information relevant to classification (such as age at onset, early treatment history, autoantibody testing, or clinician rationale) is not consistently available or summarised in the discharge documentation. In this study, most false positives corresponded to insulin-treated T2DM, while a smaller number were attributable to other causes (diabetes secondary to pancreatectomy, MODY, cystic fibrosis–related diabetes, and hyperglycaemia without diabetes), which is consistent with diabetes type misclassification being the predominant source of false positives.

The sensitivity analysis illustrates the effect of prevalence on predictive values. With a hospital discharge prevalence of T1DM of 0.54%, the population-level PPV derived from the observed sensitivity and specificity is low, reflecting that when prevalence is low, even a limited proportion of false positives can account for a substantial share of coded cases. In practical terms, the CMBD T1DM code can be used to identify potential T1DM cases for research, but additional verification steps may be needed when analyses require greater diagnostic certainty, particularly in older age groups. In contrast, in patients younger than 30 years, where no false positives or false negatives were observed in the sample, the findings support the use of CMBD-coded T1DM in analyses focused on younger populations.

Although not the primary objective, the descriptive profile of admissions with T1DM coding was consistent with patterns described in the literature, with a younger age distribution than the overall hospital population and a higher burden of chronic complications such as chronic kidney disease and higher comorbidity scores [5,22,23]. These descriptive results provide context for the study population but do not affect the main validation estimates.

In relation to prior evidence, several studies conducted in other settings have addressed the challenge of distinguishing T1DM from T2DM in routinely collected data by combining administrative diagnosis codes with additional information such as laboratory results and pharmacy/dispensing data [24,25]. These approaches are relevant because they illustrate the mechanisms by which diabetes type classification may be improved beyond diagnosis codes alone, particularly in settings where insulin use is common in T2DM. However, the aims and reference standards differ across studies. For example, some models or algorithms are developed to classify diabetes type using multiple data streams and may perform well for identifying cohorts and estimating prevalence or incidence, but they do not necessarily validate whether an existing administrative code (e.g., a discharge diagnosis code) correctly reflects the clinically confirmed diabetes type at the individual level [24,25]. Our findings, showing a proportion of false positives concentrated in older age groups and largely attributable to insulin-treated T2DM, are consistent with the premise that code-only identification is more vulnerable to misclassification when clinical phenotypes overlap and when the information required to distinguish diabetes type is not systematically captured in the coded dataset.

In Spain, validation research has more frequently focused on primary care electronic health records and on conditions other than diabetes, using clinical record review as the reference to assess the correctness of coded diagnoses. Examples include the validation of acute myocardial infarction and stroke diagnoses and the validation of atrial fibrillation, which reported substantial agreement when compared with medical record review [26,27]. These studies support the feasibility and value of direct, record-based validation designs in Spanish routine data systems. In the hospital setting, in contrast, CMBD validation has been more limited and has often focused on administrative fields rather than on clinical diagnoses. The study in Osakidetza reported discrepancies between CMBD variables and the medical record, with variability across hospitals and across fields, indicating that data quality can vary by variable type and organisational context [28]. Although that work did not evaluate T1DM specifically, it provides a relevant background for interpreting our results because it highlights that discordance between CMBD and clinical documentation is possible even in standardised datasets, and that diagnosis validity cannot be assumed without condition-specific assessment.

With respect to T1DM, the available Spanish hospital-based evidence includes a paediatric study in Extremadura that applied a capture–recapture approach, comparing CMBD records with insulin prescription data in a population under 15 years of age [29]. That study identified coding inconsistencies (reported as approximately 30.3% coding errors) and provided an indirect estimate of completeness by using an external data source, but it did not confirm diagnoses at the individual level using clinical criteria and did not report standard diagnostic accuracy measures such as sensitivity, specificity, predictive values, or kappa concordance [29]. The present study complements and extends that evidence by using individual-level clinical confirmation based on ADA criteria and by reporting sensitivity, specificity, predictive values, and kappa agreement overall and by age group. In addition, our stratified results suggest that validity may differ substantially by age, with specificity declining in older patients, which is not directly addressed by paediatric-only assessments and supports caution when extrapolating validity estimates across age groups, clinical contexts, or coding settings.

These findings have important implications for the future secondary use of health data in Spain and Europe. The CMBD of Hospital Clínico San Carlos will be integrated into the National Health Data Space, which is being federated with the European Health Data Space (EHDS). This integration will enable harmonised variable-level interoperability and support the development of large-scale European cohorts of patients with type 1 diabetes. Ensuring the diagnostic validity of CMBD coding is therefore essential to guarantee the reliability of future multicentre and cross-national research initiatives.

However, this study has limitations that should be considered. First, it was conducted in a single tertiary hospital, which may limit generalisability to other settings. Nevertheless, Hospital Clínico San Carlos is part of the Group 3 tertiary hospitals in the Madrid public health system, which share comparable case-mix profiles and apply the same ICD-10-ES standards and regional coding procedures, suggesting that large differences in coding performance across similar centres may be limited; in addition, the hospital serves a substantial reference population in Madrid, supporting the relevance of these findings for tertiary care in the region. Second, the cross-sectional design did not allow for the assessment of temporal variation in coding accuracy across the 2016–2023 period, which could be influenced by organisational factors such as staffing, training, or regulatory updates. Third, although the sample size was sufficient for overall estimates of diagnostic accuracy, stratified estimates—particularly in older age groups, where misclassification was more frequent—were based on smaller numbers and should be interpreted with caution. Finally, because this is a hospital discharge-based validation, the findings reflect inpatient documentation and coding practices and may not be directly transferable to community settings or primary care records.

## 5. Conclusions

The validation results indicate that T1DM diagnoses recorded in the CMBD represent a valid tool for epidemiological studies in individuals younger than 30 years. In older patients, the use of the T1DM code in the CMBD remains useful due to its high sensitivity, but additional strategies are required to confirm cases. Future multicentre studies including additional hospitals and diagnoses are warranted to confirm the broader generalisability and applicability of these findings.

This validation supports the use of CMBD-coded T1DM diagnoses in younger populations and provides a foundation for their integration into national and European health data infrastructures.

## Figures and Tables

**Table 1:** Characteristics of hospital discharges with T1DM and the validation sample with T1DM during 2016–2023.

		Discharges with T1DM Diagnosis (n = 1324)	Sample of Discharges with T1DM Diagnosis (n = 384)	*p* Values
Gender	(% females)	45.77	42.19	0.214
Age (years)	Mean (SD)	51.19 (22.60)	51.36 (22.53)	0.896
	Median (IQR)	52.37 (34.42–69.04)	51.42 (34.44–67.97)	0.982
<30	(%)	18.73	18.23	
≥30 to <40		11.4	11.46	
≥40 to <50		15.71	16.67	0.767
≥50 to <60		16.84	18.75	
≥60 to <70		13.9	11.72	
≥70 to <80		12.99	11.2	
≥80		10.42	11.98	
Length of stay	Mean (SD)	7.50 (10.61)	7.52 (11.47)	0.976
	Median (IQR)	4 (2–9)	4 (2–10)	0.638
Readmitted within <30 days	(%)	12.99	13.54	0.778
Year of hospital discharge	(%)			
2016		14.73	12.76	
2017		9.21	10.42	
2018		10.05	11.72	
2019		12.39	13.28	0.865
2020		13.37	13.02	
2021		14.27	12.24	
2022		14.95	15.36	
2023		11.03	11.2	
Type 1 DM	(%)	100	100	
Type 1 DM as main diagnosis		26.44	25.78	0.798
Dementia	(%)	1.89	2.08	0.807
Malignant neoplasm	(%)	8.38	9.11	0.652
Myocardial infarction	(%)	26.21	29.17	0.383
Cerebrovascular disease	(%)	1.89	2.6	0.843
Heart failure	(%)	8.38	5.47	0.873
Renal disease	(%)	9.37	9.64	0.250
Death	(%)	3.7	4.17	0.674
Charlson Index	Mean (SD)	3.18 (1.58)	3.30 (1.56)	0.202
	Median (IQR)	3 (2–4)	3 (2–4)	0.133

**Table 2:** Sensitivity, specificity, and concordance for type 1 DM.

	TN	FN	FP	TP	Sensitivity % (CI 95%)	χ^2^ *p*-Value	Specificity % (CI 95%)	χ^2^ *p*-Value	Diagnostic Concordance Kappa (CI 95%)	PPV (CI 95%)	NPV (CI 95%)
T1DM	384	0	95	289	100 (98.69–100)	-	80.17 (76.36–83.49)	-	0.753 (0.707–0.798)	75.26 (70.71–79.31)	100 (99.01–100)
Female	163	0	40	122	100 (96.95–100)	-	80.30 (74.28–85.18)	0.89	0.754 (0.684–0.823)	75.31 (68.13–81.31)	100 (97.70–100)
Male	221	0	55	167	100 (97.75–100)		80.07 (74.96–84.36)		0.752 (0.692–0.811)	75.23 (69.15–80.44)	100 (98.29–100)
<30 years	72	0	0	70	100 (94.80–100)	-	100 (94.94–100)	<0.001	1 (1–1)	100 (94.8–100)	100 (94.8–100)
≤30 to <40	50	0	5	39	100 (91.03–100)		91.91 (80.42–96.05)		0.892 (0.801–0.984)	88.64 (76.02–95.05)	100 (92.86–100)
≤40 to <50	57	0	5	59	100 (93.89–100)		90.91 (80.42–96.05)		0.917 (0.847–0.988)	92.19 (82.98–96.62)	100 (93.69–100)
≤50 to <60	69	0	12	60	100 (93.98–100)		85.19 (75.87–91.32)		0.830 (740–0.921)	83.33 (73.09–90.2)	100 (94.73–100)
≤60 to <70	45	0	14	31	100 (88.97–100)		76.27 (64.03–85.31)		0.689 (0.547–0.831)	68.89 (54.36–80.47)	100 (92.14–100)
≤70 to <80	43	0	27	16	100 (80.64–100)		60.43 (49.72–71.955)		0.372 (0.219–0.525)	37.21 (24.38–52.14)	100 (91.8–100)
≥80	48	0	32	14	100 (78.47–100)		60.00 (49.04–70.04)		0.309 (0.168–0.449)	30.43 (19.08–44.81)	100 (92.59–100)

TP: true positive; FP: false positive; TN: true negative; FN: false negative; CI 95%: confidence interval at 95%; PPV: Positive Predictive Value; NPV: Negative Predictive Value; T1DM: type 1 diabetes mellitus.

**Table 3:** Predictive values for type 1 diabetes for different prevalences (sensitivity analysis).

Prevalence	PPV (CI 95%)	NPV (CI 95%)
0.1%	0.5 (0.42–0.6)	100 (100–100)
0.2%	1 (0.84–1.2)	100 (100–100)
0.3%	1.5 (1.25–1.78)	100 (100–100)
0.4%	1.99 (1.66–2.37)	100 (100–100)
0.5%	2.47 (2.07–2.94)	100 (100–100)
1.0%	4.85 (4.08–5.75)	100 (100–100)
1.5%	7.13 (6.03–8.42)	100 (100–100)
2.0%	9.33 (7.91–10.97)	100 (100–100)
2.5%	11.45 (9.75–13.4)	100 (100–100)
3.0%	13.49 (11.52–15.73)	100 (100–100)
3.5%	15.46 (13.25–17.96)	100 (100–100)
4.0%	17.36 (14.93–20.1)	100 (100–100)

PPV: Positive Predictive Value; NPV: Negative Predictive Value; CI 95%: confidence interval at 95%.

**Table 4:** Positive predictive values (PPV, 95% CI) of type 1 diabetes mellitus coding in hospital discharge records, stratified by age group and simulated prevalence scenarios.

Prevalence/ PPV (CI 95%)	<30	30–40	40–50	50–60	60–70	70–80	>80
0.1%	100 (100–100)	1.09 (0.48–2.48)	1.23 (0.53–2.8)	0.67 (0.4–1.13)	0.42 (0.27–0.66)	0.26 (0.19–0.35)	0.25 (0.19–0.33)
0.2%	100 (100–100)	2.16 (0.95–4.84)	2.43 (1.06–5.45)	1.34 (0.8–2.23)	0.84 (0.53–1.32)	0.52 (0.39–0.69)	0.5 (0.38–0.65)
0.3%	100 (100–100)	3.2 (1.42–7.09)	3.6 (1.59–7.96)	1.99 (1.19–3.31)	1.25 (0.8–1.96)	0.77 (0.58–1.04)	0.75 (0.57–0.97)
0.4%	100 (100–100)	4.23 (1.88–9.25)	4.74 (2.1–10.35)	2.64 (1.58–4.37)	1.66 (1.06–2.61)	1.03 (0.77–1.38)	0.99 (0.76–1.3)
0.5%	100 (100–100)	5.24 (2.34–11.31)	5.87 (2.62–12.62)	3.28 (1.97–5.41)	2.07 (1.32–3.24)	1.29 (0.96–1.72)	1.24 (0.95–1.62)
1.0%	100 (100–100)	10 (4.6–20.4)	11.13 (5.13–22.5)	6.38 (3.89–10.31)	4.08 (2.62–6.3)	2.55 (1.91–3.4)	2.46 (1.89–3.2)
1.5%	100 (100–100)	14.35 (6.77–27.87)	15.88 (7.54–30.44)	9.32 (5.75–14.77)	6.03 (3.9–9.21)	3.8 (2.85–5.04)	3.67 (2.83–4.74)
2.0%	100 (100–100)	18.33 (8.87–34.12)	20.2 (9.85–36.97)	12.11 (7.55–18.85)	7.92 (5.16–11.96)	5.03 (3.79–6.64)	4.85 (3.76–6.26)
2.5%	100 (100–100)	22 (10.9–39.41)	24.13 (12.07–42.42)	14.75 (9.31–22.59)	9.75 (6.4–14.58)	6.23 (4.71–8.2)	6.02 (4.67–7.74)
3.0%	100 (100–100)	25.39 (12.85–43.97)	27.72 (14.2–47.05)	17.27 (11.02–26.03)	11.53 (7.62–17.08)	7.42 (5.63–9.73)	7.18 (5.58–9.18)
3.5%	100 (100–100)	28.52 (14.75–47.92)	31.02 (16.25–51.03)	19.67 (12.68–29.21)	13.26 (8.82–19.45)	8.6 (6.54–11.22)	8.31 (6.48–10.6)
4.0%	100 (100–100)	31.43 (16.58–51.39)	34.07 (18.23–54.49)	21.95 (14.3–32.16)	14.94 (10–21.72)	9.75 (7.44–12.68)	9.43 (7.38–11.99)

Negative predictive values (NPV) were omitted, as they were 100% in all scenarios analysed.

## Data Availability

The data underlying this study were derived from the Hospital Discharge Database (CMBD) of Hospital Clínico San Carlos. These data are not publicly available due to legal and ethical restrictions, as they contain confidential patient information and validation required access to individual medical records.

## Abbreviations

ADA	American Diabetes Association
CMBD	Conjunto Mínimo Básico de Datos (Spanish National Hospital Discharge Database)
EHR	Electronic Health Record
HCSC	Hospital Clínico San Carlos
ICD-10	International Classification of Diseases, 10th Revision
NPV	Negative Predictive Value
PPV	Positive Predictive Value
T1DM	Type 1 Diabetes Mellitus
T2DM	Type 2 Diabetes Mellitus
